# A Mild Rebuke

**Published:** 1891-01-31

**Authors:** 


### Passing Topics.

### A MILD REBUKE.

A medical contemporary has been administering to a certain print which poses as the friend of charitable institutions a mild and somewhat tardy rebuke for devoting the chief portion of its Christmas appeal issue to an onslaught upon two of our greatest hospitals—the London and Guy's. Time was when the dullest manager thought it necessary to produce for his patrons at Christmas time something both novel and attractive; but this sorry travestie has been played without variation for the last six months, and would not have called for even a passing notice had it not gained unmerited importance by the comments of the *Lancet*.

If we take the lucubrations of journals of this stamp seriously, we are disposed to say that the remonstrance of our contemporary is altogether inadequate to the offence. It would not be expostulation, but pulverization that would suffice. The difficulty is to get up any great head of indignation when the occasion presents features so ludicrous. On the one hand, we have publications which live by the favour of the institutions they affect to represent, and cannot boast a half-pennyworth of independent judgment among them; and, on the other, important public charities performing functions absolutely indispensable. It appears either comic or intolerable, according to the way we approach the subject, and possibly the state of our health, that publications of this sort should assume judicial duties and pretend to be so terribly in earnest.

That they have some qualms of what in more highly-developed organisms would be conscience is made evident by the way they endeavour to find support and justification in culling commendatory quotations from one another, but what they most rely upon is the reluctance to retort. There is always a disposition on the part of the really great, whether individuals or institutions, to be magnanimous, and silence, judiciously utilised, exercises enormous power. But while the reputation of our great medical charities is little likely to suffer, among those who know, by reason of this foolish parody of criticism, we have to remember that a large number of readers are willing to accept ready-made opinions, and to take as gospel truth the wildest efforts of irresponsible writers—so they be in print.

As a sample of the methods employed, we may refer to the paragraph in a recent issue, where the reader is jocularly asked whether it is true "there is a good deal of sympathy for the London Hospital among secretaries of other hospitals?" and then is told that "Some of them seem more concerned than the officials connected with it." We have no means of ascertaining if this be a true statement of the actual and comparative degree of sympathy evoked among right feeling and intelligent witnesses, but it seems likely that if the sneer should happen to be resented by those at whom it is levelled, this precious publication would fall upon evil days. If it wishes to survive let it mend its ways and "live cleanly." To persistently attack and strive to injure great and useful charities under pretence of legitimate criticism, while belauding ventures of dubious value, is not the way to invite or preserve confidence, and it may soon become a question whether respectable institutions should continue to countenance and support by their presence in the advertisement columns a performance conceived and executed without regard to good taste, or the accomplishment of any useful purpose.

Agricola.

